# COVERS Neonatal Pain Scale: Development and Validation

**DOI:** 10.1155/2010/496719

**Published:** 2010-10-05

**Authors:** Ivan L. Hand, Lawrence Noble, Donna Geiss, Laura Wozniak, Charles Hall

**Affiliations:** ^1^Division of Neonatology, Department of Pediatrics, Queens Hospital Center, Mount Sinai School of Medicine, 82-68 164th Street, Jamaica, NY 11432, USA; ^2^Division of Neonatology, Elmhurst Medical Center, Mount Sinai School of Medicine, Elmhurst, NY 11373, USA; ^3^Department of Nursing, Jacobi Medical Center, Bronx, NY 10461, USA; ^4^David Geffen School of Medicine, UCLA Medical Center, Los Angeles, CA 90502, USA; ^5^Department of Epidemiology and Population Health, Albert Einstein College of Medicine, Bronx, NY 10467, USA

## Abstract

Newborns and infants are often exposed to painful procedures during hospitalization. Several different scales have been validated to assess pain in specific populations of pediatric patients, but no single scale can easily and accurately assess pain in all newborns and infants regardless of gestational age and disease state. A new pain scale was developed, the COVERS scale, which incorporates 6 physiological and behavioral measures for scoring. Newborns admitted to the Neonatal Intensive Care Unit or Well Baby Nursery were evaluated for pain/discomfort during two procedures, a heel prick and a diaper change. Pain was assessed using indicators from three previously established scales (CRIES, the Premature Infant Pain Profile, and the Neonatal Infant Pain Scale), as well as the COVERS Scale, depending upon gestational age. Premature infant testing resulted in similar pain assessments using the COVERS and PIPP scales with an *r* = 0.84. For the full-term infants, the COVERS scale and NIPS scale resulted in similar pain assessments with an *r* = 0.95. The COVERS scale is a valid pain scale that can be used in the clinical setting to assess pain in newborns and infants and is universally applicable to all neonates, regardless of their age or physiological state.

## 1. Background

The definition of pain was established by the International Association for the Study of Pain (IASP) in 1979 as “an unpleasant sensory and emotional experience associated with actual or potential tissue damage, or described in terms of such damage” [1]. Newborns and infants are often exposed to numerous procedures during hospitalization which can be characterized as painful. In terms of requirements for pain perception, by 20 weeks gestation, the fetal neocortex is present, and pain pathways to the brain stem and thalamus are completely myelinated by 30 weeks [2].

Pain in neonates is often underrecognized and undertreated [3]. In addition, early exposure to pain has been shown to affect the way babies respond to pain later in life [4]. It is, therefore, important for clinicians to assess and manage pain on a regular basis in order to avoid excessive exposure.

Several validated and reliable pain scales exist to measure acute pain in term and preterm neonates. These scales incorporate a combination of behavioral indicators of pain (e.g., facial expression, body movements, and crying) and/or physiological indicators of pain (e.g., changes in heart rate, respiratory rate, blood pressure, oxygen saturation [SaO_2_], vagal tone, palmar sweating, and plasma cortisol or catecholamine levels) to assess pain in neonates. CRIES is a postoperative pain measurement score that includes crying, the requirement for oxygen supplementation (for SaO_2 _>95%), increases in heart rate and blood pressure, facial expression, and sleeplessness [5]. The Premature Infant Pain Profile (PIPP) is a seven-indicator composite measure that includes gestational age, behavioral state, heart rate, oxygen saturation, and facial actions (brow bulge, eye squeeze, and nasolabial furrow) [6, 7]. The Neonatal Infant Pain Scale (NIPS) is based solely on behavioral indicators of pain (facial expression, cry, breathing patterns, movements of arms and legs, and state of arousal) [8]. The Neonatal Facial Coding System (NFCS) is a unidimensional measure that includes multiple indicators of facial expression and was developed for use in pain research [9, 10].

Despite the number of pain measures that are available, no single scale can easily and accurately assess pain in all newborns and infants, especially in very low birth weight neonates or those who require mechanical ventilation. The objective of this study was to develop and validate a single pain scale (the COVERS scale) as a measure that can be used clinically to assess pain in newborns and infants regardless of their gestational ages and disease states. 

## 2. Methods

### 2.1. Study Participants

Study participants were 21 newborn infants admitted to the Neonatal Intensive Care Unit at Jacobi Medical Center. Infants with congenital anomalies, severe neurological abnormalities or who had received pain medications within 12 hours of the evaluation were excluded from this study. The gestational age of infants ranged from 27–40 weeks. Informed parental consent was obtained, and the study was approved by the Institutional Review Board.

### 2.2. Protocol

In order to be able to assess the needs of all infants, including the extremely low birth weight, sedated, and/or ventilated infants, the previously established scales were modified. The structure of the new scales incorporated many of the measures of previous scales but the measures were redefined, more descriptors were included, and a category called “signaling distress” was added. With these changes, the COVERS scale was developed (Table 1). The COVERS scale is based on six different physiological and behavioral measures, each with a possible score of 0, 1, or 2 for a maximum score ranging from 0 to 12. The physiological measures include changes in heart rate, blood pressure, and respiratory rate. The behavioral indicators include facial expression, resting state, body movements, and crying.

### 2.3. Measures

Newborns admitted to the Neonatal Intensive Care Unit at Jacobi Medical Center were evaluated for pain during two procedures, a heel stick and a diaper change. All infants were eligible for the study, including those who were premature, very low birth weight, intubated, and/or recovering from surgery, provided they did not meet any exclusion criteria as cited above. The procedures used for measuring pain were all part of the infants' routine hospital care and occurred within the same 12-hour period. A crossover design was used so that each patient included in the study was assessed during both procedures. 

A single observer rated pain at the patients' bedside at three different time points: at baseline (before any handling or interventions had taken place), during the procedure (heel stick or diaper change), and after a recovery period (during which no handling or interventions had taken place). The pain responses were initially measured using a composite scale that incorporated indicators from the three previously established pain scales (CRIES, PIPP, and NIPS) as well as the COVERS scale. The indicators were later separated and analyzed in accordance with their appropriate scales so that the COVERS scale could be compared to the already validated scales.

### 2.4. Data Analysis

To establish concurrent validity, scores on the COVERS scale were compared to the PIPP and NIPS, for premature and full-term infants, respectively. To establish construct validity, scores on the COVERS scale for each of the two procedures were compared. Data was analyzed using Pearson correlation coefficients and the Wilcoxon signed-rank test.

## 3. Results

Pain scores were measured for 21 newborn infants, 57% male, 0–80 (mean 22.6) days old, with gestational age ranging from 27–40 (mean 34.9) weeks. Of the 21 newborn infants included in the study, 13 were premature (<37 weeks) and 8 were full term. Demographic data for the patients is included in Table 2.

**Figure 1:**
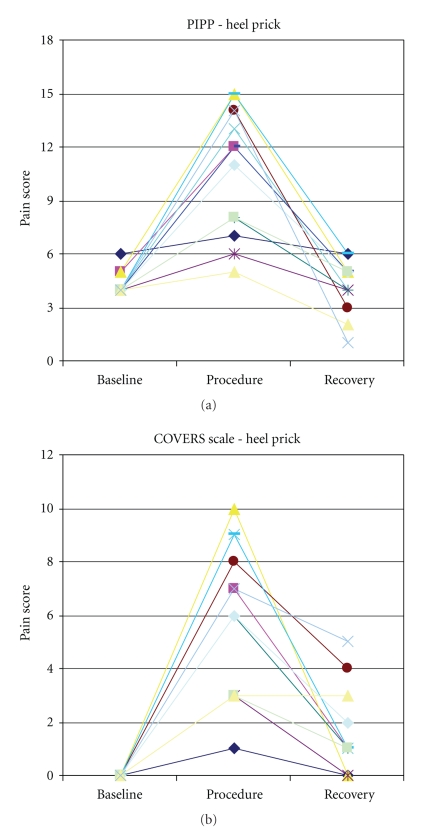
Pain scores for premature infants during the heel stick.

**Figure 2:**
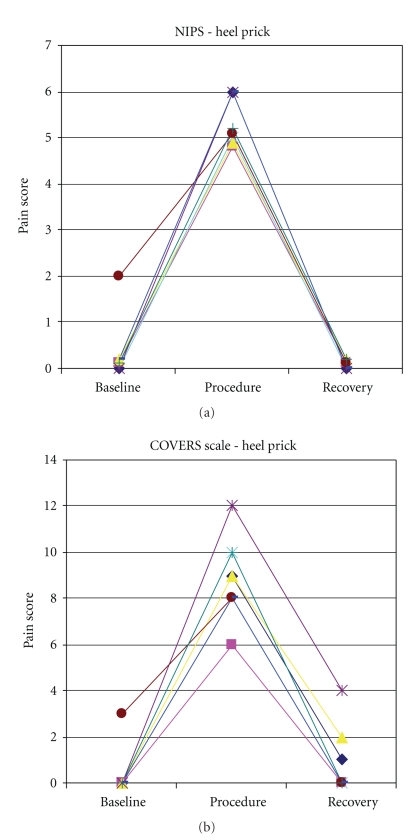
Pain scores for full-term infants during the heel stick.

In order to establish concurrent validity, the COVERS scale scores for the premature infants were compared to the PIPP scores, while those for the full-term infants were compared to the NIPS scores. For the premature infants, the COVERS scale and PIPP scale resulted in similar pain scores with an *r* = 0.84 (Table 3, Figure 1). For the full-term infants, the COVERS scale and NIPS scale resulted in similar pain scores with an *r* = 0.95 (Table 4, Figure 2).

**Figure 3:**
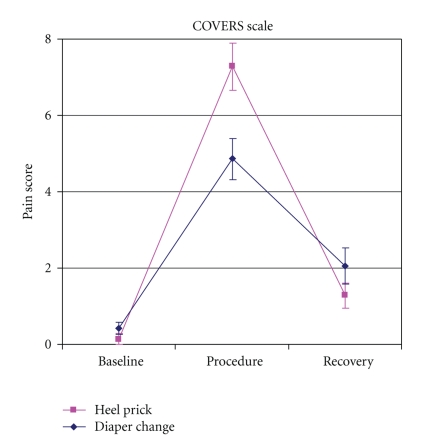
Mean COVERS Scores for all infants during the heel stick and diaper change (*n* = 13).

In order to establish construct validity, the COVERS scores for the “painful” heel stick procedure were compared to those for the “nonpainful” diaper change procedure (Table 5, Figure 3). There was no significant difference between the pain scores at baseline for the heel stick (range 0–3, mean 0.1) and diaper change (range 0–2, mean 0.4). During both procedures, the pain score had a significant increase from baseline (*P* < .05). For the heel stick, the scores ranged from 1–12 with a mean of 7.3. For the diaper change, the scores ranged from 0–10 with a mean of 4.9. In addition, the pain rating during the heel stick was significantly greater than during the diaper change (*P* < .05). After the recovery period, there was a significant decrease in the mean pain score for both procedures (*P* < .05). However, there was no significant difference between the pain scores after recovery for the heel stick (range 0–5, mean 1.3) and diaper change (range 0–8, mean 2.0). 

## 4. Conclusions

The results of this study demonstrate that the COVERS scale is a valid pain scale that can be used in the clinical setting to assess pain in newborns and infants. Concurrent validity is defined as the extent to which a test yields the same results as other measures of the same phenomenon. Concurrent validity was established by comparing the COVERS scale to previously validated pain scales, namely the PIPP and NIPS, and demonstrating a high degree of correlation. Construct validity is defined as the extent to which a test measures what it is intended to measure. Construct validity was established by comparing the pain scores on the COVERS scale during a “painful” and “nonpainful” procedure and demonstrating a significant difference between values.

The COVERS scale is an easy-to-use scale that addresses pain assessment in a broad range of newborn infants. The CRIES scale (though noted for its ease of use) has limited usefulness in measuring pain in the intubated, paralyzed, or extremely premature infant. The PIPP is best suited for preterm infants, has some subjectivity, and is complicated to score. The NIPS does not include any physiological parameters (HR, BP, and O_2_ requirement) which are often early indicators of pain and/or distress in premature, sedated, or paralyzed infants. 

Research has shown that neonates react to pain with a combination of behavioral and physiologic responses. Similar to other previously validated pain scales, the COVERS scale is multidimensional and thus incorporates these responses. The unique feature of the COVERS scale is that the criteria used for scoring are applicable to a wider range of infants. High-pitched crying is one of the behavioral responses to pain, but an intubated infant physically cannot make such a cry, which creates a dilemma for the caregiver assessing pain. The COVERS scale takes this into account by including visible crying as a behavioral response. The scale also addresses oxygen requirements from a new perspective. Rather than recording the infant's oxygen requirement, which is not always indicative of pain, it looks at a change in the need for oxygen. This increases the COVERS scale applicability to infants who may be intubated or on supplemental oxygen at baseline. One limitation that the COVERS scale does have is that it cannot be used to assess paralyzed infants since they cannot perform behavioral responses such as crying, grimacing, or signaling distress. However, the scale also incorporates physiological responses that would still apply to paralyzed infants. 

Another important aspect of a pain scale is its acceptability to the medical staff that will be using it. It has already been shown that CRIES was well accepted and in fact preferred by nurses [5]. The COVERS scale retained much of the ease of use of the CRIES, and should also be well received in clinical situations. Further study is necessary to determine if the COVERS scale is perceived as easy to use as well as to validate interrater reliability and applicability to infants beyond the newborn period.

This paper has demonstrated that the COVERS scale has both concurrent and construct validity and is thus a valid pain scale that can be used in the clinical setting to assess pain in newborns and infants. In comparison to other previously validated pain scales, the COVERS scale has the clinical advantage of being universally applicable to all neonates, regardless of their age or physiological state. This includes infants who are premature, very low birth weight, intubated, and/or recovering from surgery. It is well established that neonates perceive, respond to, and remember pain. It is, therefore, essential that pain be assessed and managed in this patient population. With the use of the COVERS scale, medical and nursing staff can gain a better grasp of the pain and discomforts their patients are experiencing as well as means to verify that attempts at managing pain in this population are successful.

## Figures and Tables

**Table 1:** COVERS Scale.

	0	1	2
Crying	No	High pitched or visibly crying	Inconsolable or difficult to soothe
Oxygen requirement	None	<30%	>30%
	At baseline O_2_	↑<20%	↑>20%
	Breathing comfortably	Change in breathing pattern	Significant change in breathing pattern

Vital signs	HR &/or BP WNL for age or at baseline	HR &/or BP ↑<20% of baseline	HR &/or BP ↑>20% of baseline
	No apnea or bradycardia or at baseline	↑ in frequency of apnea & bradycardia	↑in frequency and severity of apnea & bradycardia

Expression	None/facial muscles relaxed	Grimace, min-mod brow bulge, eye squeeze, nasolabial furrow	Grimace/grunt, mod-max row bulge eye squeeze, nasolabial furrow

Resting	Sleeping most of time	Wakes at frequent intervals—fussy	Constantly awake (even when not disturbed)

Signaling distress	Relaxed	Arms/legs flexed or extended, “time-out signals”	Flailing, arching

**Table 2:** Demographic characteristics of the sample.

Patient	Sex	Days of life	Gestational age (weeks)
1	M	19	32
2	M	5	40
3	M	55	34
4	M	80	35
5	M	20	34
6	M	12	32
7	F	10	33
8	M	65	39
9	M	26	32
10	F	58	32
11	F	47	39
12	M	8	37
13	M	23	34
14	F	9	27
15	F	3	31
16	M	19	40
17	F	3	39
18	F	2	33
19	F	9	29
20	F	0	40
21	M	2	40

	Males 12	Mean 22.6	Mean 34.9
	Females 9	Range 0–80	Range 27–40

**Table 3:** Mean Scores for premature infants during the heel stick (*n* = 13).

	Baseline	Procedure	Recovery
PIPP	4.3	10.8	4.2
COVERS	0	6.2	1.5

**Table 4:** Mean Scores for full-term infants during the heel stick (*n* = 8).

	Baseline	Procedure	Recovery
NIPS	0.3	5.4	0
COVERS	0.4	9	0.9

**Table 5:** Mean COVERS Scores for all infants during the heel stick and diaper change (*n* = 13).

	Baseline	Procedure	Recovery
Heel stick	0.1	7.3	1.3
Diaper change	0.4	4.9	2.0
	*P* > .05	*P* < .05	*P* > .05
